# Withdrawal: Novel role for p21-activated kinase 2 in thrombin-induced monocyte migration

**DOI:** 10.1016/j.jbc.2022.101879

**Published:** 2022-04-11

**Authors:** Ravisekhar Gadepalli, Sivareddy Kotla, Mark R. Heckle, Shailendra K. Verma, Nikhlesh K. Singh, Gadiparthi N. Rao

This article has been withdrawn by the authors. The Journal states that the immunoblots corresponding to ‘Rac1’ in figure 4B and 4H were duplicated. The immunoblots corresponding to ‘beta-Tubulin’ in figure 4B and ‘Pak2’ in figure 5E were duplicated. Possible micrograph reuse for ‘RhoA ASO3 + Thrombin’ panel in figure 4C and ‘Control ASO’ panel in figure 10D. Additional potential reuse in ‘Pak2 ASO2 + Thrombin’ panel in figure 5C and ‘G-alpha-qASO2’ panel in figure 10D. Possible reuse in immunoblot panels for ‘GTP-RhoA’ in figure 4E and ‘GTP-Rac1’ in figure 10J. Additional potential reuse between micrograph panels ‘SCH79797+Thrombin’ in figure 8A and ‘Control ASO’ in figure 2C. There was possible image reuse between immunoblots ‘beta-Tubulin’ panel in figure 7A and ‘Pak2’ panel in figure 7F. Finally, there was possible image reuse for ‘PY20’ immunoblot panel in figures 7D and 9E, which represent different experimental conditions. Due to these immunoblot and image duplications, the authors wish to withdraw this article.

